# Acetabular Wall Weakening in Total Hip Arthroplasty: A Pilot Study

**DOI:** 10.3390/pathophysiology30020008

**Published:** 2023-03-23

**Authors:** Madeline Gautreaux, Steven Kautz, Zashiana Martin, Edward Morgan, R. Shane Barton, Matthew Dubose, Hayden McBride, Giovanni F. Solitro

**Affiliations:** Department of Orthopedic Surgery, LSU Health Shreveport, 1501 Kings Hwy, Shreveport, LA 71103, USA

**Keywords:** arthroplasty, hip, acetabular strength, rim fracture

## Abstract

Total hip arthroplasty is a widely performed operation allowing disabled patients to improve their quality of life to a degree greater than any other elective procedure. Planning for a THA requires adequate patient assessment and preoperative characterizations of acetabular bone loss via radiographs and specific classification schemes. Some surgeons may be inclined to ream at a larger diameter thinking it would lead to a more stable press-fit, but this could be detrimental to the acetabular wall, leading to intraoperative fracture. In the attempt to reduce the incidence of intraoperative fractures, the current study aims to identify how increased reaming diameter degrades and weakens the acetabular rim strength. We hypothesized that there is proportionality between the reaming diameter and the reduction in acetabular strength. To test this hypothesis, this study used bone surrogates, templated from CT scans, and reamed at different diameters. The obtained bone surrogate models were then tested using an Intron 8874 mechanical testing machine (Instron, Norwood, MA) equipped with a custom-made fixture. Analysis of variance (ANOVA) was used to identify differences among reamed diameters while linear regression was used to identify the relationship between reamed diameters and acetabular strength. We found a moderate correlation between increasing reaming diameter that induced thinning of the acetabular wall and radial load damage. For the simplified acetabular model used in this study, it supported our hypothesis and is a promising first attempt in providing quantitative data for acetabular weakening induced by reaming.

## 1. Introduction

With the increasing aging population, higher rates of diagnosis of advanced arthritis, and a growing demand for improved mobility and quality of life, the number of people undergoing total hip arthroplasty (THA) is on the rise with the largest demographic seen in patients over the age of 70 [1]. As this procedure becomes more popular, the patient population for THA is also changing, with more young and active patients opting for THA due to osteonecrosis, osteoarthritis, and juvenile idiopathic arthritis [2]. Factoring in the increasing aging population and change in age demographics of THA, recent data have shown primary total hip arthroplasty is projected to grow 71%, to 635,000 procedures by 2030 [3]. In 2021 alone, over one million total hip and knee arthroplasties were performed in the United States [4]. Even though this is a highly successful operation, the number of revision procedures are also expected to increase each year especially in the younger population [5]. Joint registries have noticed a gradual increase in 10-year revision rates in patients under 60 compared to the older population [6]. Early failures in THA, leading to revision, are commonly caused by infection, dislocation due to aseptic loosening, osteolysis, wear and intraoperative fracture during primary THA [7]. Periprosthetic joint infection (PJI) is a catastrophic complication following THA due to challenging management involving multiple interventions and the prolonged use of antibiotics impacting the patient’s quality of life [8]. In a study by Kelmer et al., information from 535 patients with THA revisions was collected to determine the various mechanisms of failure in primary THA. They found early revisions within two years after primary THA, labeled as early revisions, are more likely to be a result of intraoperative fracture which occurred in approximately 11% of revision cases. It is likely early revisions are due to implant positioning, patient comorbidities, sterilization technique, and antibiotic prophylaxis [9]. Early migration, which is measured two years post-operatively, is a well-established predictor for the late aseptic loosening of primary acetabular components and can assess the performance and wear of the implants [10]. Aseptic loosening, defined as failure of the fixation of the prosthetic component in the absence of infection, is estimated to cause approximately 19% of late THA revisions [11]. Risk factors for this instability are multifactorial and can be patient specific, including age, gender, and abductor deficiency, or related to operative variables, e.g., femoral head diameter, component malposition, and surgical approach [12]. This late failure of THA is most commonly due to long term wear of the implant articulations used, such as metal on polyethylene or metal on metal, but more durable implants have been developed to prevent these issues and to decrease the need for revision THA [9].

Preoperative planning for THA requires radiographic review as well as the identification of anatomical bone landmarks, implant position, and size with templating [12]. To perform THA, surgeons must undergo extensive training during residency and fellowship after completion of medical school. Physicians first complete five years of residency with strict oversight from the American College of Graduate Medical Education (ACGME) which ensures they are proficient in all aspects of orthopedic surgery. Subsequently, orthopedic hip surgeons complete an additional year of training to learn the complex skills, latest techniques, and technology, learning how to handle complex hip pathologies, including revision surgery [13].

The press-fit principle for cup fixation, adopted in 1996, is a widely used technique for THA where a hemispherical socket is placed in an under-reamed acetabulum. This principle is highly accepted due to no screw usage, no leakage of polyethylene powder out of the screw hole, and relatively easy operation at the time of the procedure [14,15]. Surgeons are able to predict the cup size for press-fit after templating is performed using two-dimensional templating. Unfortunately, predicting implant size from templating is imprecise carrying only a 16–62% correct size prediction [16]. A study performed by Brulc et al. showed the surgeon was the main independent risk factor for unsuccessful intra-operative press-fit fixation regardless of the surgeon’s yearly hip arthroplasty volume and training [17]. Achieving accurate acetabular positioning within the joint is essential to achieve function and longevity. The initial stability of uncemented press-fit components relies on rim peripheral bone contact forces induced by under-reaming. Therefore, if surgeons medialize past the supportive rim, there is an increased risk of loosening, but this risk is also high when over-lateralizing the cup due to inadequate superolateral bony support [16]. Under-reaming, determined by the surgeon and their subjective assessment of bone quality [11], is essential because a very small difference in the degree of under-reaming can substantially influence the insertion force of the fixation [11]. Most surgeons under-ream the acetabulum by 1 or 2 mm for the initial fixation [11] but other studies raise concerns for 2 mm of under-reaming due to excessive stress on the acetabulum, especially if there is osteoporosis or marked osteosclerosis, increasing the risk of fracture along with other complications. Therefore, caution should be taken to avoid fracture of the acetabular wall, especially for higher amounts of under-reaming [18]. Lack of specific indications for preoperative planning due to difficulty with exposure, problems gaining initial implant fixation, and suboptimal stability, along with factors, such as poor bone quality and use of more aggressive press-fit designs with strong impaction forces, play a crucial role in determining intraoperative and post operative problems [11].

Furthermore, larger implant diameters have been developed to decrease the incidence of instability by increasing the head/neck ratio, delaying neck/cup contact, and extending implant range of motion after THA [19]. Some surgeons advocate for the use of these larger cup implants because they allow for a larger contact area which facilitates biological fixation and strain distribution over a large area of the pelvis for a higher friction fit [20]. Primary stability of large diameter cups relies only on press-fit, as screw fixation is not possible with this diameter [21]. Literature studies have shown that the use of larger cups can increase stability by decreasing the risk of dislocation to 1% compared to the standard-head group with a dislocation rate of 8.7% [22]. However, the use of large diameter cups needs to be taken with caution according to Skeels et al. who reported a 17% dislocation occurrence in patients who received a 36–40-mm diameter head [23]. The advantages for increased cup size include avoiding structural bone grafts or reconstruction long-term and allowing for the use of larger femoral head sizes which could decrease the dislocation rate and allow for better hip kinetics long-term [24]. However, the preparation of the acetabular surface to accept an implant that is much larger than the native acetabulum requires reaming into both anterior and posterior columns and medial wall, which potentially causes weakening of the acetabular wall, which may increase the risk for intraoperative fracture [25]. Surgeon’s ream the acetabulum to the appropriate size and position based on the preoperative template and intraoperative landmarks while maintaining proper inclination and anteversion [26]. In fact, excessive reaming has been associated with increased risk of exceeding host bone strength with consequential acetabular fracture [27]. Intraoperative acetabular fracture in press-fit insertion of acetabular cups has been documented in several studies [28,29], and while a rare complication, it still occurs at a rate of 0.49%, but depends on various factors, such as the amount of under-reaming, stiffness of bone, magnitude of force applied to the cup and the size and strength of the acetabulum after it has been reamed [7]. In the attempt to reduce the incidence of intraoperative fractures, the current study aims to identify how increased reaming diameter degrades and weakens the acetabular rim strength. We hypothesized that there is proportionality between the reaming diameter and the reduction in acetabular strength.

## 2. Materials and Methods

**Figure 1:**
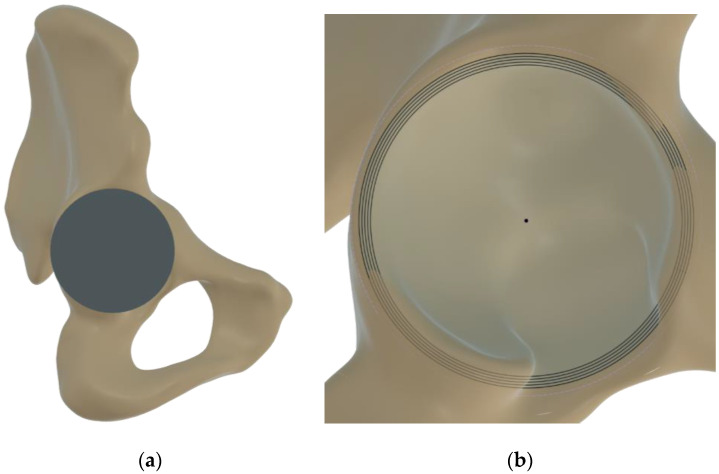
Acetabular cup templating (**a**) and reaming of concentric layers in 1 mm diameter increments (**b**).

After receiving Institutional Reviewer Board approval, the computer tomography (CT) scans of human pelvises were acquired from the database of a Level 1 trauma center, excluding subjects with previous pelvic surgery, acetabular column or ilium fractures, osteoporotic bone, sclerotic bone, and hip dysplasia. The tridimensional reconstruction of the bone was performed using Slicer3d [30] and smoothed in Autodesk Meshmixer with a solid accuracy of 0.75 mm. The resultant model was then exported in Fusion360 (Autodesk, San Rafael, CA, USA) for virtual instrumentation of the acetabular cup. An orthopedic surgeon trained in total joints replacement performed acetabular cup templating through frontal and lateral views of the pelvis (see Figure 1a). Following the positioning, preserving the position of the cup apex in relation to the bone, values of reaming were imposed from 54 to 58 mm in 1-mm increments through Boolean operations (see Figure 1b).

**Figure 2:**
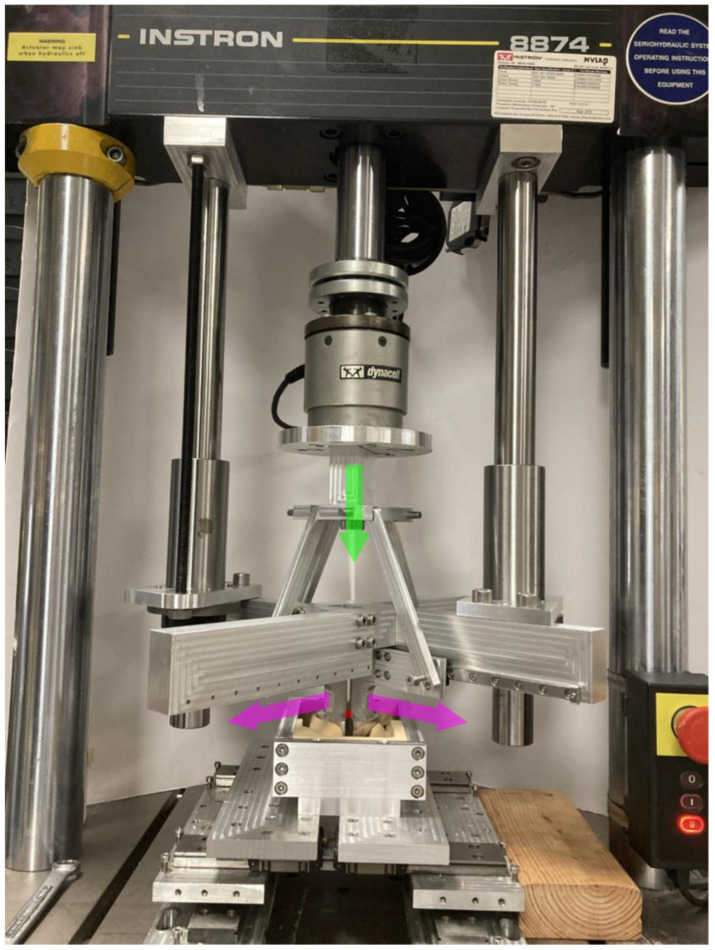
Custom made fixture used to convert the axial displacement of the Instron actuator (shown in green) in the radial expansion of the acetabulum (shown in purple) with reference to the center of the templated cup (shown in red).

The obtained pelvic models were then machined out of 20 pounds per cubic foot (PCF) (0.32 g/cm^3^) solid rigid polyurethane foam (Sawbones, Vashon Island, WA, USA) as previously adopted for pelvis models in mechanical testing [31]. The obtained bone surrogate models were then tested using an Intron 8874 mechanical testing machine (Instron, Norwood, MA, USA) equipped with a custom-made fixture. The latter was made by a rigid frame connected to the Instron crosshead that hosts four THK low friction rails (THK Co., Ltd., Tokyo, Japan) disposed radially in the plane parallel to the plane of the templated cup. The linear guides were centered on the templated cup and each of them allowed the radial displacement in the cup plane of a rigid arm shaped as the quarter portion of a hemispherical cup. The simultaneous expansion of these quarter portions was actuated through rigid brackets connected to the Instron actuator. This mechanism converted the axial movement in the radial expansion of the acetabulum in correspondence of the templated acetabular cup (see Figure 2). The expansion was imposed while preserving the position of the cup center that was also kept the same among all the considered reaming diameters.

During expansion, the load at which was generated a disruption of the acetabular ring was extracted as the damage load of reference. Three repetitions were performed for each configuration for a total of 15 experiments. Analysis of variance (ANOVA) was used to identify differences among reamed diameters, while linear regression of the damage loads in relation to the nominal reamed diameter was used to evaluate the potential relationship between the acetabular strength and the reamed diameters.

## 3. Results

**Figure 3:**
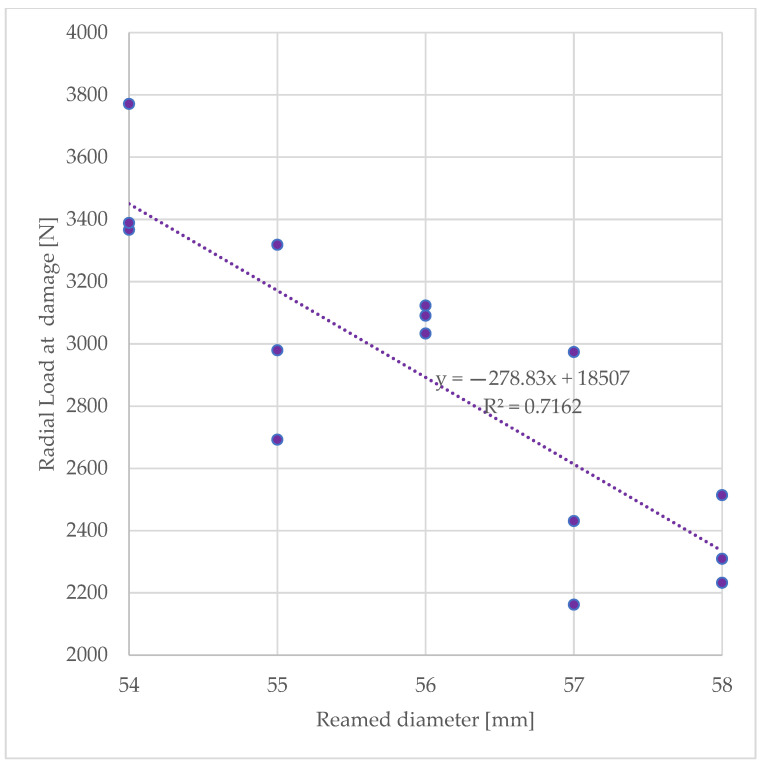
Experimentally determined acetabular strength expressed in as the peak radial force measured during radial expansion.

Radial load at rim damage was affected by the reamed diameter (*p* = 0.01) and was measured to be 3508 N ± 227 for the smallest reamed diameter of 54 mm and reduced to a value of 2352 N ± 146 for the largest reamed diameter of 58 mm. Increased reaming diameters that induced a thinning of the acetabular wall were found moderately correlated to the radial load at damage (R^2^ = 0.716, see Figure 3)

## 4. Discussion

Reducing or preventing complications will be of great importance in the emerging healthcare environment based on quality control and patient outcomes [32]. A previous retrospective study on 745 patients who underwent THA showed that 39% of patients underwent revision THA within only five years after primary THA. Of the 39%, 33% were revised due to instability, 30% for aseptic loosening, 14% for painful hemiarthroplasties, 14% for infection, 5% for wear-related failure, and 3% for periprosthetic fractures. The same study did notice a downward trend of revision for aseptic loosening over the years showing there is a better understanding of the technical requirements needed for lasting results [33]. Currently, no studies show a significant association between age and instability post-revision, but Badarudeen et al. showed a significant association between instability and female gender, which demonstrated a greater risk of 71% compared to males [34]. Revision surgery only accounts for approximately 10% of the surgical volume of hip arthroplasties but are associated with more complications and higher mortality, resulting in greater costs, causing an economic burden [35]. After reviewing the total hospital cost for revision surgery, revision implants, due to the complex nature of revision components, contribute a major aspect of the overall cost [36].

We have found that incrementing the reaming diameter results in a degradation and thinning of the acetabular wall in terms of its capability in withstanding radial acetabular loads. Furthermore, we have identified a moderate correlation between the load at which the loss of continuity in the acetabular ring and the reamed diameters is generated. As with most of the pilot studies, the current study has several limitations that we will overcome, extending the methodology proposed here on cadaveric specimens. We used bone surrogates, as is commonly done in the field of orthopedic biomechanics research when more than two configurations need to be tested to address a particular research question [37,38,39]. Beckmann et al. used more than two configurations in order to determine which of the three tested fixation techniques, i.e., cement-only, screw- only, and combination of cement and screw, provided the most stable bond between the porous titanium acetabular component and augment [37]. Huber and Noble used multiple configurations to examine the fixation traits of a six-finned acetabular cup in both primary and revision THA and then compared it to two commonly used cups without fins [38]. The use of 20-pounds-per-cubic-foot (pcf) density bone surrogates for acetabular cup stability has also been previously successfully done by [11,31,40], where all chose this density due to the high success rates in previous studies. Goossens et al. designed a study using bone surrogates to investigate the relationship between relative bone-implant micromotions and the more commonly used load-to-failure implant stability metrics. This study was conducted to provide better insight during the preclinical testing of new acetabular cup designs, new surgical procedures, and to provide a useful tool for surgical training [40]. Hickernell et al. also used bone surrogates to compare initial shell stability under different reaming techniques with HS (hemispherical) and NHS (nonhemispherical) acetabular components [11]. Schieriott et al. used bone surrogates to test the effect of bone defects and bone defect filling on the primary stability of the press-fit model to provide a platform to test and compare different treatment strategies for increasing bone defect severity in a standardized way [31].

In the current study, we do not have implanted acetabular cups but simulated the expansion needed to accommodate the cup with under-reaming conditions. This technique is clinically executed in 1-mm diameter increments. However, in the current study, the expansion was continuous. Therefore, we are not able to draw conclusions regarding the under-reaming value that is safe to use for each acetabular wall thinning considered in the study. This methodological approach has been firstly used by Amirouche et al., in 2017, to try to understand how a reduction in pelvic bone mineral density affects the oversizing of the prothesis for primary THA and determine if the location of the segmental defect affected cup fixation while considering an experimental validation previously published [18,41]. While the experiments were carried out without an axial loading of the cup, it must be noted that this approach allowed us to maintain our study not confined to a particular reaming value. An additional limitation is that we have limited our focus on the implant–bone interface not accounting for the technology used in the coupling with the femoral component. The reduction in dislocation rates for dual mobility cups that has been documented [25] may be instrumental in reducing the dimensions of the needed reamed diameter.

Furthermore, another relevant limitation is that the study is performed on an acetabulum without defects or osteoporotic bone. Although similarities exist between the different classification systems, each one has a unique grading scale ranging from mild to severe defects [42]. Therefore, bone structure must be analyzed and classified based on bone loss by a combination of preoperative AP overview, additional information about the anterior and posterior acetabular columns, computer tomographic imaging, and ultimately intraoperative findings of the bony configuration of the acetabulum [43]. The AAOS classification system has gained wide acceptance and is able to distinguish between segmental and cavitary defects, allowing for the practical, simplified assessment of bone defects and preoperative evaluation of acetabular defects, in turn enabling the proper preparation of surgical approach and determination of the necessary implants [42]. The Paprosky classification system is based on the presence or absence of supporting structures, such as the acetabular rim, superior dome, medial wall, anterior and posterior columns, and the surgeons assessment of these structures’ capacity to support the prosthesis [44]. Acetabular defects are concomitant to reduced bone density [21] and when limited to Paprosky defects Type I and II, it is advised that over-reaming may result in a greater risk of acetabular wall weakening [43]. When fixing acetabular defects characterized as type I and IIA defects, press-fitting uncemented components via underreaming relies on a rim of peripheral cortical bone for their initial stability but will suffice [16]. For pelvises characterized by low bone density or presence of acetabular defects, strategies other than oversizing should be chosen as noted in previous studies [43].

## 5. Conclusions

Previous studies have analyzed reaming in relation to cup size in terms of insertion forces and micromotion, but susceptibility of the acetabulum to the chosen parameters before implantation has remained overlooked. In conclusion, the current study represents a first attempt to provide quantitative data on previously indicated acetabular weakening induced by the reaming. The finding on a simplified acetabular model supports what has been previously hypothesized. Therefore, the extension of the methodology here proposed to cadaveric pelvises will allow the translation of these findings in clinically relevant reaming values.

## Data Availability

Not applicable.
